# Amino Acid Interactions (INTAA) web server v2.0: a single service for computation of energetics and conservation in biomolecular 3D structures

**DOI:** 10.1093/nar/gkab377

**Published:** 2021-05-21

**Authors:** Jiří Vymětal, David Jakubec, Jakub Galgonek, Jiří Vondrášek

**Affiliations:** Institute of Organic Chemistry and Biochemistry of the Czech Academy of Sciences, Praha 6, 160 00, Czech Republic; Institute of Organic Chemistry and Biochemistry of the Czech Academy of Sciences, Praha 6, 160 00, Czech Republic; Department of Software Engineering, Faculty of Mathematics and Physics, Charles University, Praha 1, 118 00, Czech Republic; Institute of Organic Chemistry and Biochemistry of the Czech Academy of Sciences, Praha 6, 160 00, Czech Republic; Institute of Organic Chemistry and Biochemistry of the Czech Academy of Sciences, Praha 6, 160 00, Czech Republic

## Abstract

Interactions among amino acid residues are the principal contributor to the stability of the three-dimensional structure of a protein. The Amino Acid Interactions (INTAA) web server (https://bioinfo.uochb.cas.cz/INTAA/) has established itself as a unique computational resource, which enables users to calculate the contribution of individual residues in a biomolecular structure to its total energy using a molecular mechanical scoring function. In this update, we describe major additions to the web server which help solidify its position as a robust, comprehensive resource for biomolecular structure analysis. Importantly, a new continuum solvation model was introduced, allowing more accurate representation of electrostatic interactions in aqueous media. In addition, a low-overhead pipeline for the estimation of evolutionary conservation in protein chains has been added. New visualization options were introduced as well, allowing users to easily switch between and interrelate the energetic and evolutionary views of the investigated structures.

## Graphical Abstract

**Graphical Abstract:**
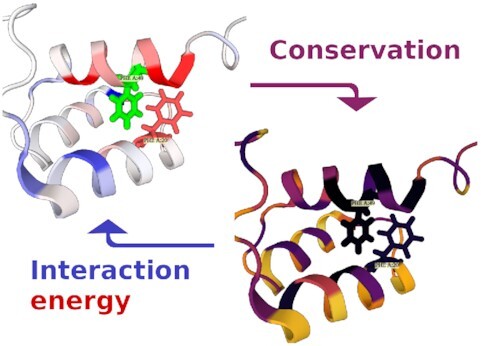
The Amino Acid Interactions (INTAA) web server is a unique computational resource which newly allows its users to interactively inspect both energetic and evolutionary properties of biomolecular structures.

## INTRODUCTION

The stable arrangement of amino acid residues known as the three-dimensional (3D) structure of a protein is the result of various covalent and noncovalent interactions among the residues as well as interactions with the environment, i.e. the molecules of the solvent. The overall stability of a protein can be quantified as the Gibbs free energy difference between the folded and unfolded states. It has been experimentally shown that, in many proteins, only a fraction of residues is essential for maintaining their 3D structure and function while the rest can be mutated without any major effect on stability (1).

Amino acid residues critical for protein stability can be identified and analyzed using, for example, the theoretical methods of computational chemistry and biophysics. These residues typically participate in noncovalent interactions characterized by highly favorable interaction energies (IEs). The IEs of individual amino acid residue pairs can be arranged in the form of a square interaction energy matrix (IEM), which has been shown to be a powerful tool for the identification of structurally and functionally significant residues (1,2).

In addition to the physical, energetic view of the biomolecular structure, evolutionary conservation can be an important indicator of the residues’ structural and functional significance. For example, amino acid residues involved in hydrophobic core formation or enzymatic activity usually display lower substitution rates compared to other sites on the protein (3). Evolutionary conservation, quantified using, e.g. the information content (IC) of individual columns in a multiple sequence alignment (MSA) of related sequences, can thus provide information orthogonal to the analysis of IEs. IC-based mark-up can be obtained for protein 3D structures using web applications such as ConSurf (3) and 3DPatch (4); however, bridging the energetic analyses and the projections of conservation has so far not been possible to do using a single web server.

The Amino Acid Interactions (INTAA) web server (https://bioinfo.uochb.cas.cz/INTAA/) is a unique computational resource which focuses on the investigation of energetics of interactions among the constituting moieties (e.g. amino acid residues) of a biomolecular 3D structure (5). The web server's functionality is split into two services: the IEM web application, and the amino acid–nucleotide interactions web server. The latter of these components provides a static analysis of the geometric and energetic preferences in the interactions between amino acids and nucleotides in a predefined set of protein–DNA complexes. As this component has not received any major updates in this release of the INTAA web server, we will further focus on the IEM web application and refer to it interchangeably using the INTAA acronym.

The IEM web service is an interactive application which accepts as its input a biomolecular 3D structure [specified using a Protein Data Bank (PDB) (6) identifier (ID) or uploaded as a file by the user] and calculates the pairwise IEs among its constituting moieties. As described below, the query structure is automatically processed by the web server, hydrogen atoms are optionally added, and the IEs are calculated using a molecular mechanical force field (FF). The user can further choose a model used to simulate the environment as a dielectric continuum. The IEM web service then calculates for each residue in the query structure the pairwise IEs for its interactions with all other residues in the structure, as well as its total (net) IE, defined as the sum of the former. The pairwise and total IEs can be viewed numerically in the form of interactive tables, as well as shown projected onto the query structure in the integrated molecular structure viewer.

The INTAA web server has been used recently, for example, for the investigation of effective pairwise potentials between amino acid residues in proteins (7), for the comparison of the strength of interactions of particular amino acid residues (8), or as a reference method for the calculation of IEs (9). The INTAA web server has been further employed in the design of peptidic cysteine protease inhibitors targeted against the malaria parasite (10), for the elucidation of the structure–function relationship in a cyanobacterial Mn-catalase enzyme (11), and for the investigation of a missense mutation in a human UDP-glucose dehydrogenase linked with developmental delay (12).

In this update, we describe the major functions added to the INTAA web server since its original release. Notably, a new continuum solvation model has been introduced as the default option for the treatment of electrostatic interactions in aqueous media, providing a more accurate representation of the interactions. A new pipeline for the calculation of evolutionary conservation of amino acid residues in the protein chains has been developed and added to the web server. The pipeline is completely automatic and is invoked with each new task. New selection and visualization options have been further added, allowing the users to easily interrelate the energetic and evolutionary views of the query structures. These additions help establish the INTAA web server and the IEM web service as comprehensive tools for the interactive analysis of biomolecular 3D structures. In the following sections, technical description of the new features and examples of their usage are provided.

## MATERIALS AND METHODS

### New continuum solvation model

The IEM web service calculates the IE as a sum of the noncovalent van der Waals and point-charge electrostatic interactions. The electrostatic term can be evaluated using Coulomb's law, neglecting any environmental effects, or the interaction with the solvent can be introduced in an implicit manner. In the previous version of the application, the implicit solvent was modeled using a distance-dependent dielectric constant (DDDC). Although such treatment of the solvent decreases the magnitude of the electrostatic term at large distances, it was an *ad hoc* approach without solid physical grounding. Simplicity, ease of implementation, and zero performance overhead were the only advantages of this approach. On the other hand, the Poisson–Boltzmann (PB) theory (13) provides proper description of continuum electrostatics. However, solving the corresponding electrostatic field equations requires numerical integration over discretized space elements, making it time-consuming and sensitive to implementation details. Furthermore, the nonadditive character of the PB theory does not allow a straightforward separation of the pairwise interaction terms which are the cornerstone of the IEM method.

In order to improve the quality and accuracy of the electrostatic term evaluated in the dielectric continuum, the generalized Born (GB) model was adopted in the current version of the application. We have shown in the past that empirical potentials along with the GB model are able to reproduce the results of quantum chemical DFT-D/PCM calculations while being fast enough to be used for calculations involving large biological systems (14). The GB theory is the limit case of the PB theory obtained under several approximations (15). Near complete match between the GB and PB theories can be achieved when the Born radii—the critical parameters of the GB model—are calculated exactly (16). The Born radius of an atom expresses its effective distance from the dielectric boundary and hence its exposure to the solvent. Nevertheless, estimation of the Born radii is the most approximative and heuristic part of the GB theory applications. The algorithm newly implemented in the IEM web service follows the approach of Onufriev, Bashford and Case, namely the parametrization II (OBC II) described in (17). As the GB model takes the solvent exposure of the atoms into account explicitly, it clearly surpasses the accuracy of models which utilize a DDDC. In addition, the GB model permits the pairwise treatment of the electrostatic contributions compliant with the ideas of the IEM web service, and is thus well suited to serve as its default method for the calculation of electrostatics interactions. It should be noted for completeness that, when the GB model is used, the IEs provided by the IEM web service constitute effective energies affected by the implicit solvent; more specifically, while the IEs remain pairwise separable, they are affected by the calculated Born radii, which depend not only on the configuration of the residue pair, but also on the global geometry of the biomolecular 3D structure.

Implementation of the GB model has required the extension of the computational pipeline to allow for the Born radii calculation. The calculation is performed after the atom types and FF parameters had been assigned, since the radii are atom type-specific. The required parameters have been adopted from the GROMACS simulation suite (18) for all supported FFs.

### Information content (conservation) calculation

After selecting a biomolecular structure, the PDB file is processed using the Bio.PDB module (19) from the Biopython package (20) and sequences of its individual chains are read. The polypeptide chains are then identified, and a MSA of similar sequences is constructed for each using the *phmmer* tool from the HMMER software package (http://hmmer.org/). The *phmmer* search uses default parameters and UniProtKB/Swiss-Prot (21) as the target database. The target database will continue to be updated and its current version can be found in the IEM web service manual. For each MSA, the respective weights of its constituting sequences are calculated using the Gerstein/Sonnhammer/Chothia algorithm (22) as implemented in the *esl-weight* miniapp included with the HMMER software. Finally, the per-residue IC is calculated using the *esl-alistat* miniapp, taking the individual sequence weights into account.

Unlike ConSurf (3), the conservation calculation pipeline does not attempt to reconstruct the phylogenetic tree or model the sequence evolution, relying instead on the mentioned sequence-weighting algorithm to reduce the effects of uneven phylogenetic representation bias. Together with performing only pairwise alignments of the identified similar sequences against the query, this leads to a massive speed-up of the calculations, with most queries having the IC values calculated in a matter of seconds.

The IC values range between 0.0 bits (no conservation) and approx. 4.32 [log_2_(20)] bits (complete conservation). Please be aware that large IC values are also occasionally exhibited by the chains' terminal residues, as these can remain unmatched in the MSAs. Identification of such artifacts may be enabled in the future with the utilization of the respective MSA per-column gap frequency values.

The conservation calculation pipeline is implemented as a Python script and is publicly available at https://github.com/davidjakubec/INTAA-conservation under a permissive license. The pipeline is invoked automatically for each new task started by the IEM web service in parallel with the IE calculations and, due to the speed of the HMMER software and the size of the target database, constitutes at worst only a negligible slowdown of the overall computations.

### New visualization options

**Figure 1.**
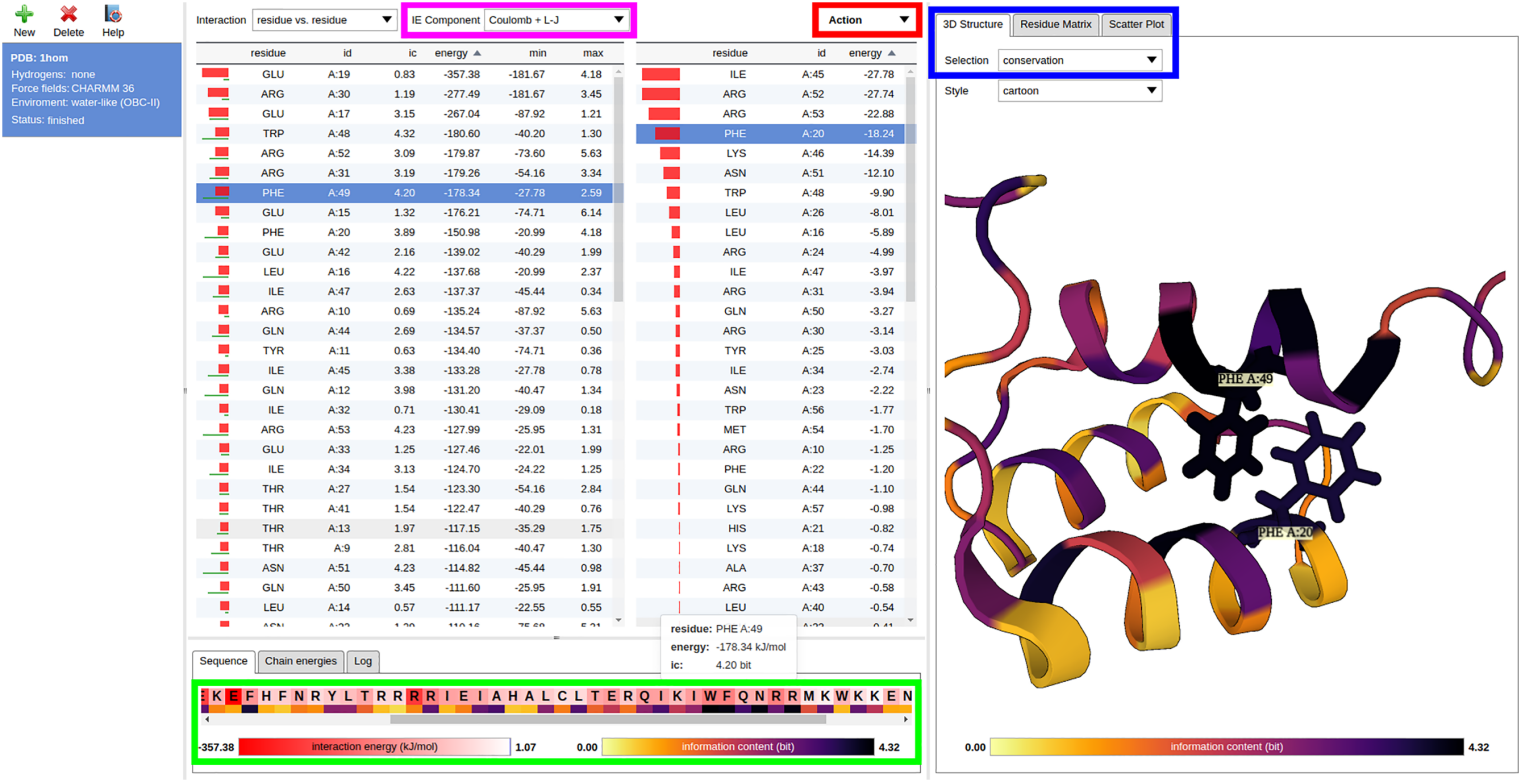
The 3D structure of the *Antennapedia* homeodomain from *Drosophila* (PDB ID 1HOM) analyzed using the IEM web service. As the hydrogen atoms were already present in the structure, none needed to be added. The IEs were calculated using the CHARMM36 FF (25) and the new OBC-II continuum solvation model. The central section of the UI features two tables of IE values. In the table on the left, the amino acid residues have been sorted according to their total IEs, with the ones displaying the most energetically favorable interactions at the top [the residues can be sorted according to any of the displayed features as described in the previous publication (5)]. The IC values calculated for each residue using the new conservation calculation pipeline have been added to this table. Once a residue is selected (in this example, Phe49), the table on the right side shows the pairwise IEs for its interactions with the other residues in the structure. These IEs can be sorted as well and the most favorable pairwise interactions can thus be identified for each residue. In this example, Phe20 is selected in the table on the right. It can be seen that the pairwise noncovalent interaction between the two selected residues is among the most energetically favorable. The integrated molecular structure viewer in the right section of the UI displays the investigated structure in cartoon representation; any residues selected within the central tables are highlighted in stick representation. The most notable additions to the web service and UI updates are highlighted in bold rectangles. The top magenta rectangle highlights the new *IE Component* drop-down menu. Here, the user can choose between the display of the Coulomb, Lennard-Jones, or complete (Coulomb + Lennard-Jones) IEs in the application. The bottom green rectangle highlights a window relating the primary sequence, the one-dimensional (1D) IE profile, and the 1D conservation profile for the residues in the structure. The window is interactive and enables the IE and IC values for individual residues to be displayed numerically. The top right blue rectangle highlights the selection boxes for the new visualization options. The *Selection* drop-down menu in the *3D Structure* tab allows the user to switch between total IE-, pairwise IE-, or conservation-based mark-ups for the investigated structure. In this example, *conservation* is selected, and the per-residue IC values are projected onto the structure. It can be seen that the two selected Phe residues are involved in the hydrophobic core of the homeodomain and are both highly conserved, in agreement with the known evolutionary properties of the domain family (24). Switching to the *Residue Matrix* tab allows the user to visualize and interact with the IE and distance matrices; switching to the *Scatter Plot* tab allows the user to interrelate the total IEs and IC values for the selected residues using an interactive 2D plot. These are described in greater detail in Figures 2 and 3, respectively. Finally, the top red rectangle highlights the new *Action* drop-down menu. This menu provides access to advanced IEM export options, configuration options, as well as a button for providing feedback to the developers.

In the current version of the application, the IC values for individual amino acid residues have been added numerically to the results table, and are also being visualized in the first column of the table using green bars (Figure 1). The current version also adds a simple sequence visualization window (*Sequence* tab) in which individual residues are colored according to their respective total IEs and IC values. The sequence visualization window is interactive and allows details about individual residues to be displayed by hovering the mouse cursor over them (Figure 1).

The integrated molecular structure viewer allows the visualized 3D structure to be annotated with an IC-based mark-up by selecting the option *conservation* from the *Selection* drop-down menu (Figure 1).

**Figure 2.**
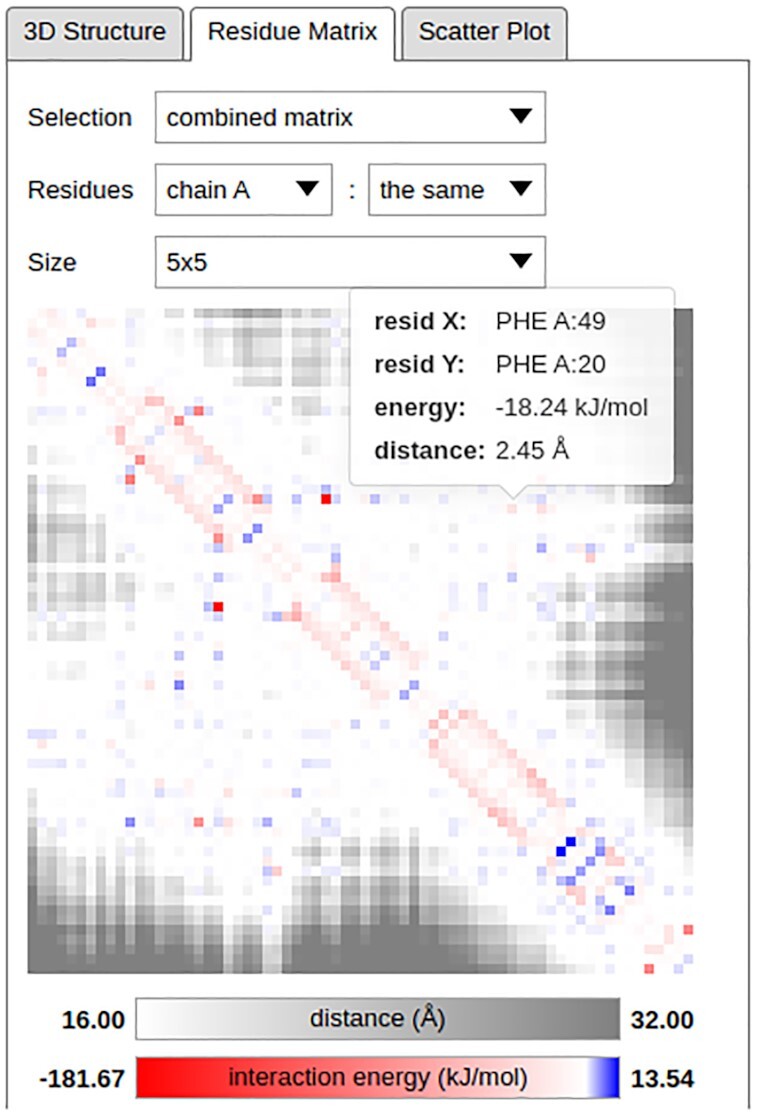
Detailed view of the IE and distance matrices corresponding to the structure investigated in Figure 1. The *combined matrix* has been chosen for visualization in the *Selection* drop-down menu, meaning that IEs are shown in the matrix viewer for residue pairs with any pair of atoms at or below a distance threshold and distances are shown for residue pairs further apart. An alternative, COM-based distance definition can be toggled using the *Configure* dialog in the new *Action* drop-down menu (Figure 1); the thresholds can be configured using this menu as well. The investigated structure contains only a single chain and the *Residues* drop-down menu selections thus lead to a symmetric matrix being displayed. Finally, the *Size* drop-down menu can be used to adjust the dimensions of the matrix elements. In this example, the Phe49–Phe20 pair investigated in Figure 1 has been highlighted in the matrix viewer, and the IE for its pairwise interaction and the closest distance between the residues’ atoms are shown.

In the current version of the application, the pairwise IE as well as residue distance matrices can be visualized in-browser using the *Residue Matrix* tab by selecting the options *energy matrix* or *distance matrix*, respectively, in the *Selection* drop-down menu (Figures 1 and 2). The distance between a pair of residues can be calculated as either the closest distance between any pair of their atoms, or as the distance between the centers of mass (COMs) of the considered moieties; these options can be toggled using the *Configure* dialog in the new *Action* drop-down menu (Figure 1). The residue distance calculations reflect the subgroup selection in the *Interaction* drop-down menu; for example, when the IEs are calculated only for the interactions among amino acid backbone atoms, only these are considered in the residue distance calculation. Finally, the in-browser matrix viewer can also visualize a combination of pairwise IEs and residue distances by selecting the option *combined matrix*. In this mode, residue pairs with mutual distance below a threshold are colored according to the pairwise IE, while pairs further apart are shown masked in greyscale. The motivation behind this mode is that the IEs for the interactions among distant residues are usually close to zero and are thus hidden due to being uninformative. The matrix viewer is interactive and allows information about the pairwise IEs and residue distances to be shown by hovering the mouse cursor over the individual residue pairs (Figure 2).

The in-browser matrix viewer can also visualize the pairwise IE and residue distance matrices for only selected parts (e.g. chains) of the investigated structure. These can be specified individually for the matrix’ rows and columns using the *Residues* drop-down menus. The size of the matrix elements can be configured using the *Size* drop-down menu (Figure 2).

The color scale limits and selected thresholds for the respective viewers utilized in the *3D Structure* and *Residue Matrix* tabs can be newly configured using the new *Action* drop-down menu (Figure 1).

**Figure 3.**
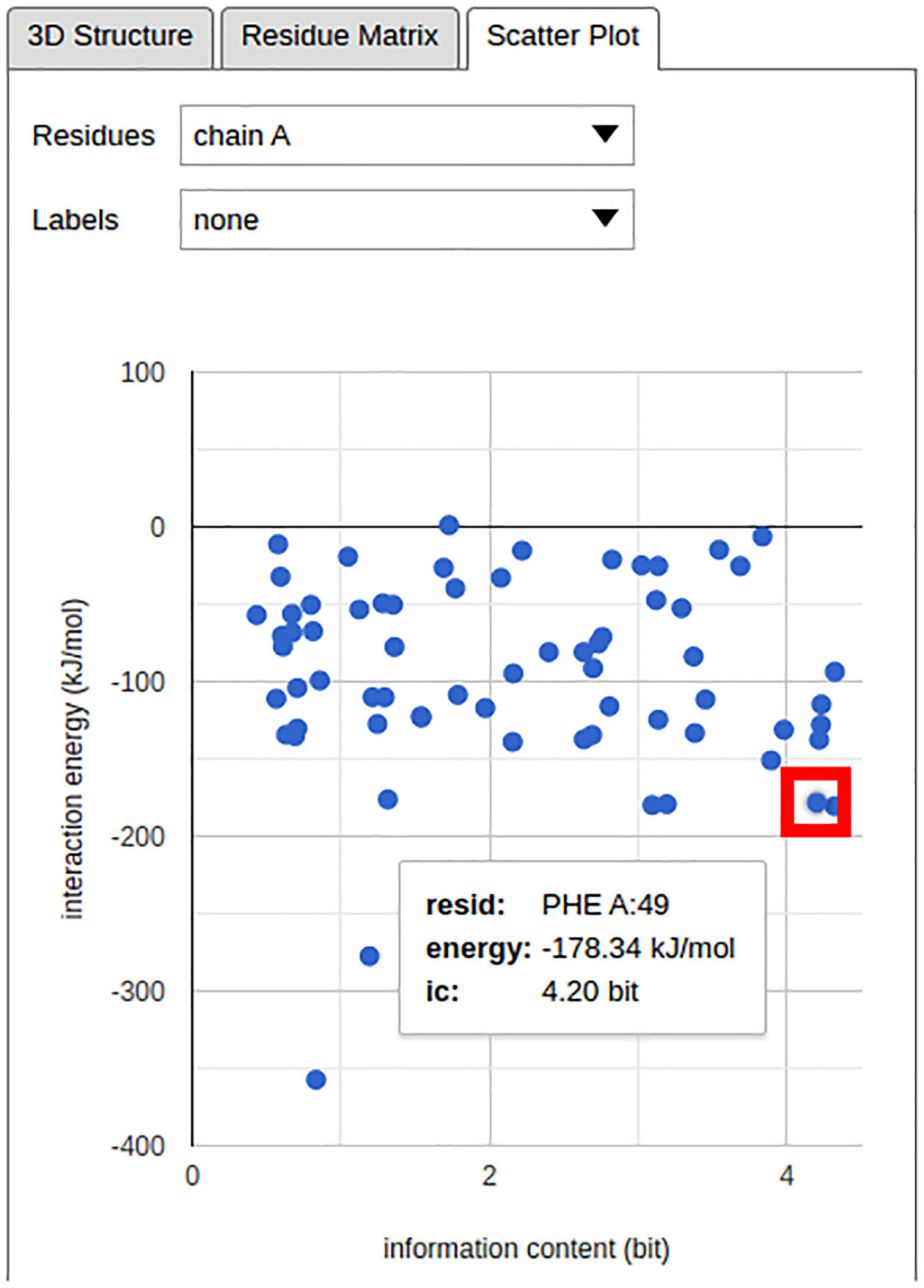
The total IE–IC value relations for the residues in the structure investigated in Figure 1. The single chain in the investigated structure has been selected using the *Residues* drop-down menu; the *Labels* drop-down menu can be used to add textual identifiers to individual points in the plot. In this example, the position of Phe49 in the scatter plot has been highlighted in a red rectangle, and its total IE and IC value are shown.

Finally, a new *Scatter Plot* tab has been added, providing a simple way to interrelate the total IEs and IC values for the selected residues in the form of an interactive two-dimensional (2D) plot (Figure 3).

### Other changes and improvements

A new *IE Component* drop-down menu has been added, allowing the user to choose between the display of the Coulomb, Lennard-Jones, or complete (i.e. Coulomb + Lennard-Jones) IEs (Figure 1). Next, the front page of the INTAA web server has received a major graphical overhaul which improves its clarity and highlights the most important information. In addition, every element in the IEM web service user interface has been extended with an informative tooltip which provides instantaneous access to its detailed description. The tooltips are accessible by simply hovering the mouse cursor over the respective elements. Furthermore, the user's choices for the drop-down menu selections are newly remembered across sessions. Finally, the on-line manual page of the IEM web service has received a major update to its content and structure and now also includes illustrative examples of the application's usage.

## RESULTS

We demonstrate the new functionality of the INTAA web server and the types of analyses it enables using an example task featuring the 3D structure of the *Antennapedia* homeodomain from *Drosophila* deposited in the PDB under the ID 1HOM (23). The structure of this DNA-binding protein domain was determined using nuclear magnetic resonance spectroscopy in solution and constitutes a suitable demonstrative example due to its small size and well-known structure–function relationship and conservation profile (24). The analysis of this structure enabled by the INTAA web server is shown in Figure 1. This Figure reviews selected elements of the IEM web service and illustrates how novel information about the relationship between energetic and evolutionary roles of specific residues can be obtained with the utilization of the new web server features. Figure 2 follows this analysis with a detailed view of the new, interactive IE and distance matrix visualization for the investigated structure. Finally, Figure 3 shows the total IE–IC value relations for all residues in the investigated structure in the form of a new, interactive 2D scatter plot visualization.

## DISCUSSION

As described above, the GB model introduced into the IEM web service in this update improves the quality and accuracy of the IE calculations. Nevertheless, the significance of the IE calculations can still depend on the quality of the user's input. Two major sources of input-related errors are known at this time. First, errors in the macromolecular structures, such as missing nonhydrogen atoms and unrealistically close atomic contacts, can result in missing or unphysical elements in the IEM. These propagate into other quantities, such as total IEs. Second, unrealistic atomic overlaps and other errors can also result from the use of tools utilized to add the missing hydrogen atoms which are necessary for the IE calculations. Both of these issues could be remedied by additional processing of the investigated structures, including modeling of its missing parts and subsequent geometry optimization. However, these procedures are difficult to implement and to perform automatically and would likely become major sources of errors or unstable behavior themselves. In addition, they would significantly increase the computational demands of the application and introduce an undesirable delay between the task submission and presentation of the results. Therefore, we decided to omit these steps and encourage the users to carefully inspect the biomolecular structures for potential errors before utilizing the IEM web service.

In this update, we have described major additions to the INTAA web server and, in particular, to the IEM web service. The utilization of the GB model makes the IE calculations in aqueous media more physically accurate. The addition of the conservation score calculations makes it, for the first time, possible to study the residues’ physical and evolutionary roles within the biomolecular structures using a single web service. The INTAA web server remains aimed for use by bioinformaticians as well as biochemists and, as illustrated in this work, the new features enable it to be utilized for a wide range of tasks in biomolecular structure analysis and engineering which benefit from interrelating the physical and evolutionary views of the structures.

## DATA AVAILABILITY

The INTAA web server is publicly available at https://bioinfo.uochb.cas.cz/INTAA/. The source code for carrying out the IC calculation tasks is available at https://github.com/davidjakubec/INTAA-conservation.

## Notes

Present address: David Jakubec, Department of Software Engineering, Faculty of Mathematics and Physics, Charles University, Praha 1, 118 00, Czech Republic.
